# Latent profiles of compassion fatigue among clinical nurse preceptors and their associations with work alienation

**DOI:** 10.1038/s41598-025-33648-6

**Published:** 2026-01-23

**Authors:** Yuejun Huang, Han Zhang, Yu Chen, Zhaojun Xu

**Affiliations:** Intensive Care Unit, Ningbo No. 2 Hospital, Ningbo, Zhejiang China

**Keywords:** Clinical nurse preceptors, Compassion fatigue, Latent profile analysis, Work alienation, Health care, Health occupations, Psychology, Psychology, Risk factors

## Abstract

This study aimed to investigate the latent profiles of clinical nurse preceptors (CNPs)’ compassion fatigue (CF), identify the influencing factors, and examine their association with work alienation. Between July and August 2025, 340 nurse preceptors from a tertiary grade A general hospital in Zhejiang Province were recruited as participants using convenience sampling. The Chinese version of the Professional Quality of Life Scale Version 5 (ProQOL-5) and the Work Alienation Scale (WAS) were used to assess compassion fatigue and work alienation, respectively. Demographic information was also collected from the participants. Latent Profile Analysis (LPA) was employed to identify potential profiles of compassion fatigue. After screening variables through univariate analysis and multicollinearity tests, multinomial logistic regression was used to assess the influencing factors. Furthermore, a one-way ANOVA was conducted to examine differences in work alienation among different potential profiles, and the results were interpreted based on the job demands-resources (JD-R) model theoretical framework. A total of 320 CNPs were included in the final analysis. The findings of the latent profile analysis indicated that three latent profiles of CNPs’ compassion fatigue were identified: high-satisfaction-low-exhaustion group (*n* = 56, 17.5%), moderate compassion fatigue group (*n* = 160, 50%), and severe exhaustion group (*n* = 104, 32.5%). Multinomial logistic regression analysis showed that age, marital status, education, years of preceptorship, experience, employment type, and professional title were significant predictors of compassion fatigue among CNPs. There were statistically significant differences in the work alienation scores among the three latent profiles (*P* < 0.001). CNPs’ compassion fatigue can be categorised into three types, with significant heterogeneity observed among them. Notable differences exist in work alienation among CNPs with different compassion fatigue types. These findings suggest that clinical managers and educators should develop targeted interventions and support systems based on these circumstances. Therefore, formulating such management strategies is crucial for alleviating work alienation among CNPs and will help improve nurse retention rates and the quality of clinical education.

## Introduction

With the continuous growth in demand for medical services, healthcare professionals are facing increasing psychological pressure in their clinical work. Nurses are the primary caregivers in the healthcare industry, and their responsibilities necessitate a comprehensive understanding of patient care. They witness patients’ fear, helplessness, stress, and traumatic experiences during clinical care^1^. Among nurses, Clinical Nurse Preceptors (CNPs) represent a critical yet particularly vulnerable subgroup. CNPs are senior nurses in clinical settings responsible for guiding nursing students. Their core task is to cultivate the professional skills and ethics of nursing students through teaching real medical scenarios^2^. CNPs take on dual roles: they must provide nursing care to patients while also carrying out the teaching responsibilities of training nursing students—duties that go beyond the typical scope of regular nurses. In addition, CNPs are expected to provide practical examples of professional conduct and clinical procedures for learners. While engaging in clinical teaching, their constant exposure to others’ suffering, combined with role overload, increases their vulnerability to compassion fatigue. If these emotions are not released in a timely manner, they can ultimately lead to compassion fatigue (CF)^3^.

The notion of CF was first proposed by Joinson^4^. It denotes the continuum of physical, emotional, spiritual, and social transformations that occur when nurses engage in prolonged empathy with patients, especially when the accumulated stress from this empathy exceeds their endurance and they lack sufficient social support^5^. This phenomenon can manifest as emotional exhaustion, professional helplessness, and depersonalization, leading to a decreased ability to empathize—a state often termed “the cost of caring.”^6^ According to a survey, 54.5% of nurses experienced CF^7^, while another study indicated that the incidence of CF among psychiatric nurses could be 73.6%^8^, demonstrating that compassion fatigue constitutes a significant challenge to occupational health within the nursing profession. This issue constitutes a global occupational health concern. A recent meta-analysis synthesizing international evidence confirms that high prevalence rates of CF are a consistent finding among nursing populations across diverse healthcare systems, including those in North America and Europe. ^9^

The psychosocial factors behind the patterns of compassion fatigue among CNPs can be clarified through theory-driven studies. and systematically identified the key determinants of heterogeneous subgroups. The job demands-resources model (JD-R model) is a theoretical framework widely used in the field of occupational psychology and was proposed by Demerouti et al. in 2001^10^. The model divides working conditions into two main categories: job demands and resources. Job demands refer to aspects of work that require sustained physical or psychological effort and may lead to health problems, such as emotional demands or role conflict. Work resources refer to factors that help achieve work objectives, reduce the negative impact of demands, or promote personal growth, such as social or team support. According to the JD-R model, job demands are mainly related to the exhaustion component of burnout (e.g., emotional exhaustion), while job resources are associated with disengagement. This finding has been empirically validated across multiple occupational groups. From the theoretical perspective of the JD-R model, compassion fatigue among CNPs can be understood as the result of a long-term imbalance between “high job demands” and “limited job resources.” CNPs shoulder the dual responsibilities of clinical nursing and teaching supervision, which significantly increases their job demands compared to general nurses: in addition to routine nursing duties, they must also demonstrate procedures in real time, answer students’ questions, assess learning outcomes, and bear the patient safety risks that may arise from students’ mistakes^11^. Understanding the adverse effects of compassion fatigue is vital to elucidating its effects on nurses’ occupational health and clinical practice. Compassion fatigue can erode empathic capacity, leading to erosion of nurses’ physical and mental health status. If left unresolved, ongoing compassion fatigue may cause CNPs to feel that leaving their profession is their only way to cope^12^. Based on the JD-R model, compassion fatigue, as a manifestation of prolonged energy depletion, may exhaust nurses’ psychological resources, thereby predisposing them to negative attitudes towards their work, such as work alienation and ultimately turnover. Thus, within the JD-R framework, CF is not merely an individual outcome but a key process that may predispose CNPs to broader negative work attitudes.

Additionally, work alienation is defined as frustration, negative emotions, and indifferent behaviors that arise when employees’ material and psychological needs are not met by the work environment^13^. Clinical nurses mainly work in shifts, face a high risk of infection, and have a heavy workload. Clinical nurses constitute one of the most susceptible groups for this psychological condition. A study indicated that approximately 87.3% of nurses experience moderate or higher levels of work alienation^14^. Chinese nurses reported moderate to high levels of work alienation, according to recent research^15^. More and more evidence indicates that clinical nurses’ work alienation can reduce their job autonomy, decision-making participation and the quality of care^16^. It may even lead to negative emotions, job burnout, and a tendency to quit^17^. Comprehending the intersection of CF with work alienation and its propensity to exacerbate it is essential to maintain a stable and competent nursing workforce.

Currently, most studies primarily rely on total scale scores to describe the overall profile of compassion fatigue among general clinical nurses. Few studies have focused on CNPs, thus failing to capture the diversity within this group and easily overlooking individual differences. Moreover, systematic research on how compassion fatigue relates to work alienation remains scarce, and whether distinct subgroups of compassion fatigue exert differential effects on work alienation behaviors has yet to be explored. Reducing the level of compassion fatigue among CNPs not only helps stabilize the nursing workforce and decrease turnover rates but also improves the quality of nursing student training, ultimately leading to better patient care outcomes.

Latent Profile Analysis (LPA) is a person-centered statistical method that identifies potential subgroups and their proportions based on individuals’ scores on observed variables^18^, thereby facilitating the examination of unique characteristics among different subgroups. In this study, this method is employed to uncover heterogeneity in compassion fatigue among CNPs, systematically analyze the distinct features and influencing factors of various subgroups, and explore their intrinsic connections with work alienation. These findings provide valuable insights for developing personalized intervention strategies, offer empirical evidence for enhancing the overall health effectiveness of the healthcare system, and promote the formulation of more population-targeted and sustainable health strategies at the intersection of occupational health and public health policies.

This study hypothesizes that (1) CNPs can be divided into several subgroups based on compassion fatigue and (2) Individual demographic characteristics influence the distribution of the subgroups. (3) there may be a positive association between the different characteristic subgroups of CNPs and work alienation. Understanding these factors can aid in precisely identifying CNP groups at high risk of CF, developing personalized intervention measures, and reducing their sense of work alienation.

## Methods

### Study design and participants

This study used a cross-sectional survey approach. From July 2025 to August 2025, a tertiary grade A general hospital in Zhejiang Province was selected as the research source by using the convenience sampling method. The numbers of registered nurses in the hospital were input into the Excel table, and 340 CNPs were selected as participants in our study. Inclusion criteria: (1) Had obtained a nurse qualification certificate; (2) Had three years or more of clinical nursing experience; (3) Volunteered for involvement in this study and provided informed consent. (4) More than one year of clinical teaching experience. Exclusion criteria: (1) Were not on duty during the investigation period, including taking leave for various reasons (sick leave, maternity leave, personal leave, annual leave), study, business trips; (2) Were non-registered on-the-job CNPs in Ningbo City; (3) Those who had recently suffered major life events or experienced emotional setbacks.

However, the generalizability of the results of this study may be limited by the research design. The use of a convenience sample from a single center means that the results may not fully represent all the CNPs. Nevertheless, this study provides valuable insights into the target population in this specific high-level medical setting.

### Ethical considerations

This study was approved by the Ethics Committee of Ningbo No. 2 Hospital (approval no. PJ-NBEY-KY-2025-155-01) and was conducted in accordance with the ethical principles of the Declaration of Helsinki. The introductory section of the online questionnaire provided all participants with the research background, aims, methods, assurance of confidentiality, voluntary participation, and the right to withdraw, thereby obtaining informed consent. Subsequently, only those individuals who agreed to participate completed the questionnaire. All collected data were anonymized and accessible exclusively to the research team.

### Data collection methods

Data were collected using the Wenjuanxing online system. A unified instruction was provided at the beginning of the questionnaire to clarify its goal, filling method, and relevant precautions. Participants could enter the formal answering interface only after they completed reading the informed consent form and clicked “Agree” .To ensure the quality of the questionnaire, this study adopted an anonymous filling method. Each WeChat ID or IP address could only participate once in the survey. According to Kendall’s sample size calculation principle, the sample size should be five to ten times the number of items in the questionnaire. Considering the loss of sample size, the sample size was increased by 10%. Therefore, at least 275 cases were required, and 340 questionnaires were distributed. After excluding questionnaires completed in less than 2 min, those with erroneous or incomplete responses, and those with a repetition rate of answer options greater than 70%, we ended up with 320 valid responses, giving us a solid 94% response rate. Finally, this study included 320 CNPs, all of whom provided informed consent.

## Measures

### Sociodemographic characteristics

The general sociodemographic characteristics table was compiled by the researchers themselves, including age, marital status, gender, educational background, years of preceptorship experience,

professional title (categorized for this study as: primary nurse or nurse-in-charge and above, based on the Chinese nursing certification system), employment type, and monthly income.

### ProQOL-5

Chinese version of the Professional Quality of Life Scale Version 5 (ProQOL-5). This scale was developed by Stamm^19^ and translated into Chinese by Chen et al.^20^ This scale consists of 30 items across three dimensions: compassion satisfaction, burnout, and secondary traumatic stress. Each dimension contains ten items. The scale adopts the Likert 5-point scoring method, with scores ranging from 1 to 5 for “ never ” to “ very often” and the total score for each of the three dimensions is 50 points. The higher the compassion satisfaction score, the greater the sense of satisfaction an individual gains. The higher the burnout and secondary traumatic stress scores, the more severe is the compassion fatigue. The critical values of the three dimensions are compassion satisfaction < 37.0 points, burnout > 27.0 points, and secondary traumatic stress > 17.0 points. When the total score of one dimension is greater than the critical value, it is considered mild compassion fatigue; moderate compassion fatigue is defined as when the sum of the scores for two dimensions is higher than the crucial value; and severe compassion fatigue is defined as when the sum of the scores for all three dimensions is higher than the critical value. The Cronbach’s α value for this scale in this study was 0.914.

### Work alienation scale

Our study employed the scale originally developed by Ren Xiaojing^21^ through empirical research. It includes three dimensions: sense of powerlessness, sense of helplessness, and sense of meaninglessness, with a total of 12 items. The scale adopts a Likert 5-level scoring method, where “Strongly Disagree” is scored as 1 point and “Strongly Agree” as 5 points, with a total score ranging from 12 to 60 points. Higher scores reflect a greater level of perceived work alienation among nurse preceptors. This study demonstrated the scale’s strong internal consistency. (Cronbach’s α = 0.844).

### Statistical methods

All data were independently extracted and double-checked by two individuals before being entered to ensure accuracy. The three dimensions of the ProQOL-5 were used for LPA to identify subgroups of compassion fatigue among the CNPs. The Akaike information criterion (AIC), Bayesian information criterion (BIC), modified Bayesian information criterion (aBIC), information entropy, LoMendell-Rubin adjusted likelihood ratio test (LMRT), and bootstrap-based likelihood ratio test index (BLRT) were used to assess the adequacy of the model. Reduced AIC, BIC, and aBIC values suggest a better fit of the model. Information entropy, which lies between 0 and 1, indicates higher accuracy in model classification as the value approaches^22^. When the value is greater than 0.8, it indicates that more than 90% of the cases are classified correctly^23^. Moreover, the differences in the k-category and k-1-category models’ fit were assessed using the LMRT and BLRT. The k-profile model demonstrated a significantly better fit than the (k − 1)-profile model (*P* < 0.05). During the selection of the optimal model, in addition to assessing various test metrics, the practical applicability and user-friendliness of the model was considered.

Data that followed a normal distribution were presented as mean ± standard deviation. Group comparisons were performed using the Kruskal-Wallis H test. Categorical variables were compared using the chi-square test. When multiple comparisons were involved, P-values and control errors were adjusted using the Bonferroni correction. In the univariate analysis, statistically significant variables were subjected to multicollinearity analysis. If the variance inflation factor (VIF) is 5 or higher, there is a high degree of multicollinearity. After removing variables associated with these issues, multinomial logistic regression analysis was used to identify the factors influencing compassion fatigue among mentoring nurse preceptors with different profiles. All variables with a P-value of less than 0.05 were chosen as independent variables in the univariate analysis and were added to the multinomial logistic regression model. we used SPSS version 29.0 software to conduct the data analysis.

## Results

### Sociodemographic characteristics of the participants

A total of 320 valid responses were collected, resulting in a valid response rate of 94%. The average age of the participants was 32.26 ± 5.94 years, and the majority were female (94.06%). Comprehensive information is presented in Table 1.

### Outcomes of latent profile analyses

In this study, we fitted five latent profile models. Based on a comprehensive comparison of the fit indices presented in Table 2 (e.g., AIC, BIC, aBIC, entropy = 0.997, and significant LMR and BLRT tests, *P* < 0.001), this study selected the three-profile model as the optimal fitting model for the latent profiles of nurse compassion fatigue. The three latent class probabilities were 17.50%, 50.00%, and 32.50% respectively.

### Named the latent profiles of clinical nurse preceptors compassion fatigue

Based on their scores on the three dimensions of compassion satisfaction, burnout, and secondary traumatic stress, nurse preceptors were assigned to one of the three latent profiles:

- Class 1 was characterized by high compassion satisfaction coupled with low burnout and low secondary traumatic stress, and was therefore named the “high-satisfaction-low-exhaustion group.”
- Class 2 displayed moderate levels on all three underlying dimensions: compassion satisfaction, burnout, and secondary traumatic stress. It was therefore named the “moderate compassion fatigue group.”
- Class 3 exhibited the highest levels of burnout and secondary traumatic stress, coupled with the lowest level of compassion satisfaction. And was therefore named the “high exhaustion group.”

**Fig. 1:**
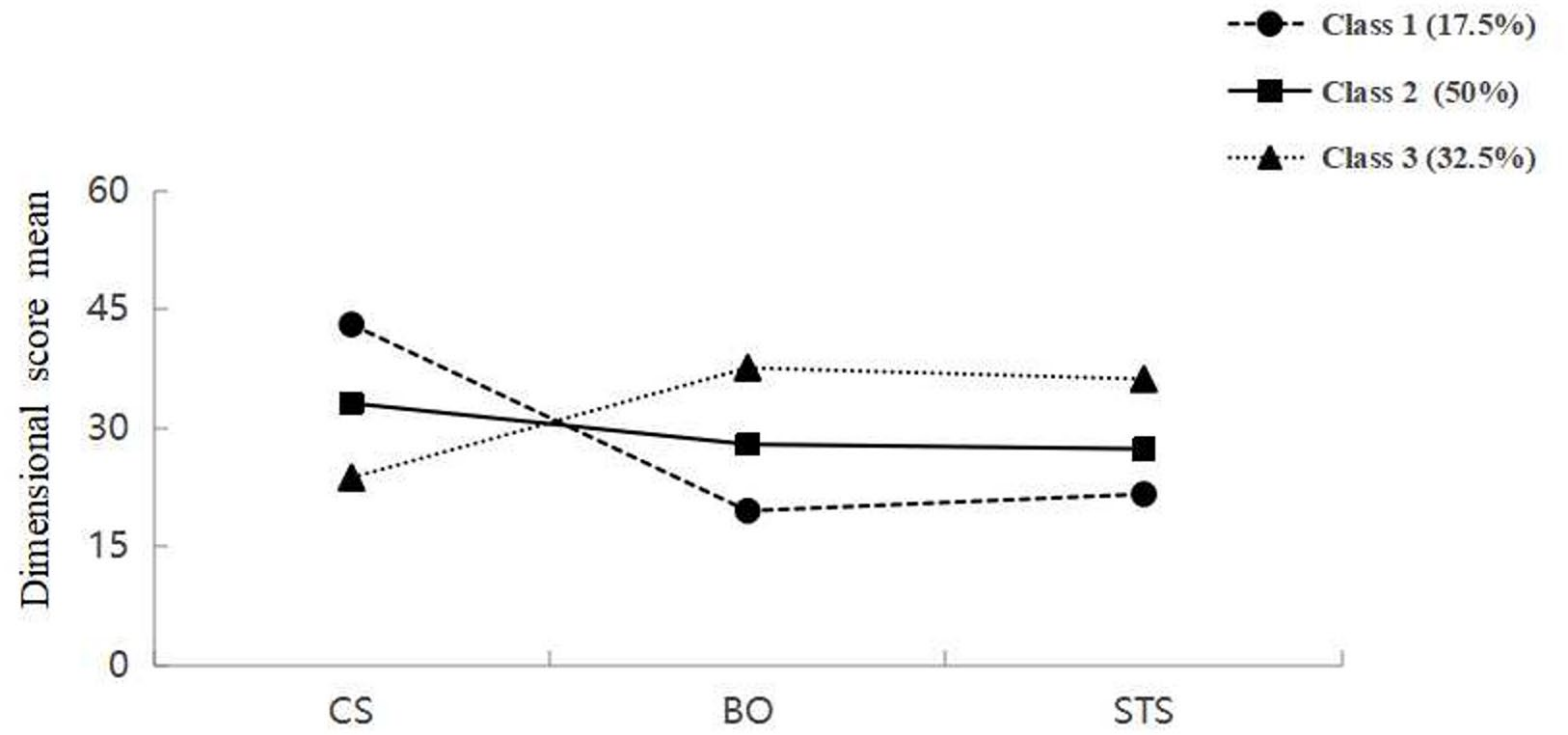
The characteristic distribution of three latent profiles of compassion fatigue. CS = Compassion Satisfaction; BO = Burnout; STS = Secondary Traumatic Stress; Class 1 = high-satisfaction-low-exhaustion group; Class 2 = moderate compassion fatigue group; Class 3 = severe exhaustion group.

These profiles are illustrated in Fig. 1. The scores across all three dimensions differed significantly among the profiles, as detailed in Table 3.

**Table 1:** Univariate analysis of the latent profile of CNPs’ compassion fatigue data are *n (%)*. a χ^2^-value. b *H*-value. class 1 = high-satisfaction-low-exhaustion group. class 2 = moderate compassion fatigue group; class 3 = severe exhaustion group. Professional titles^*^ are based on the national certification system in China. Primary nurse denotes the primary-level title, while Nurse-in-Charge and above encompasses intermediate and senior titles with greater clinical and supervisory responsibilities.

Variables	Total (*n* = 320)	Class 1 (*n* = 56)	Class 2 (*n* = 160)	Class 3 (*n* = 104)	χ^2^/H	*P*
Gender						
Male	19 (5.94)	5(8.93)	10(6.25)	4(3.85)	1.739^a^	0.419
Female	301 (94.06)	51(91.07)	150(93.75)	100(96.15)		
**Age(years)**						
≤ 29	108 (33.75)	48(85.71)	55(34.38)	5(4.81)	78.159^b^	< 0.001
30–39	176 (55.00)	6(10.71)	80(50.00)	90(86.54)		
≥ 40	36 (11.25)	2(3.57)	25(15.62)	9(8.65)		
**Years of preceptorship experience(years)**						
2–5	94(29.38)	27(48.21)	52(32.50)	15(14.42)	33.599^b^	< 0.001
6–10 > 10	144(45.00) 82(25.62)	21(37.50) 8(14.29)	80(50.00) 28(17.50)	43(41.35) 46(44.23)		
**Number of children**						
0	134(41.88)	28(50.00)	58(36.25)	48(46.15)	5.567 ^a^	0.234
1	139(43.44)	22(39.29)	73(45.63)	44(42.31)		
2	47(14.68)	6(10.71)	29(18.12)	12(11.54)		
**marital status**						
Unmarried or divorced	171 (53.44)	16(28.57)	75(46.88)	80(76.92)	39.740^a^	< 0.001
Married	149 (46.56)	40(71.43)	85(53.12)	24(23.08)		
**Education**						
Junior college	168 (52.5)	16(28.57)	70(43.75)	82(78.85)	46.718^a^	< 0.001
Bachelor’s degree or above	152 (47.5)	40(71.43)	90(56.25)	22(21.15)		
**Employment type**						
Permanent staff nurse	144 (45.00)	43(76.79)	89(55.62)	12(11.54)	77.207^a^	< 0.001
Contract dispatch or others	176 (55.00)	13(23.21)	71(44.38)	92(88.46)		
Professional title*						
Primary Nurse	152 (47.5)	40(71.43)	86(57.75)	26(25.00)	36.477^a^	< 0.001
Nurse-in-Charge and above	168 (52.5)	16(28.57)	74(46.25)	78(75.00)		
**Monthly income (RMB)**						
< 5000	54 (16.87)	9(16.07)	35(21.88)	10(9.62)	6.962^a^	0.138
5000–10,000	110 (34.38)	20(35.71)	50(31.25)	40(38.46)		
> 10,000	156 (48.75)	27(48.22)	75(46.87)	54(51.92)		

**Table 2:** Latent profile model fit indicators. LL, Log likelihood; AIC, Akaike’s information criteria; BIC, Bayesian information criteria; aBIC, sample-size adjusted BIC; LMR, Lo–Mendel–Rubin; BLRT, Bootstrapped likelihood ratio test.

Model	LL	AIC	BIC	aBIC	Entyopy	LMR *P* value		BLRT *P* value	Latent class probability
Class 1	-3132.214	6276.428	6299.037	6280.007	*	*	*		*
Class 2	-2799.042	5618.083	5655.767	5624.048	0.826	<0.001	<0.001		0.322/0.678
Class 3	-2529.775	5087.549	5140.306	5095.9	0.997	<0.001	<0.001		0.174/0.501/0.325
Class 4	-2508.454	5052.908	5120.738	5063.645	0.954	0.129	<0.001		0.094/0.173/0.502/0.226
Class 5	-2493.588	5031.176	5114.079	5044.299	0.859	0.484	<0.001		0.503/0.094/0.173/0.012/0.218

**Table 3:** Mean scores of the compassion fatigue scale across latent profiles of CNPs class 1 = high-satisfaction-low-exhaustion group; class 2 = moderate compassion fatigue group; class 3 = severe exhaustion group.

Group	Compassion Satisfaction	burnout	secondary traumatic stress	Total score
Class 1	43.09 ± 2.99	19.52 ± 2.67	21.63 ± 4.21	84.32 ± 5.38
Class 2	33.12 ± 2.11	27.93 ± 1.57	27.35 ± 2.49	88.38 ± 3.80
Class 3	23.71 ± 2.31	37.61 ± 3.19	36.18 ± 3.69	97.53 ± 5.66

### Comparison of work alienation among CNPs with different types of compassion fatigue

Based on the results of the latent feature analysis, a one-way ANOVA was conducted to examine the relationship between the three types of compassion fatigue characteristics and work alienation. The results revealed significant differences in overall work alienation scores between the three compassion fatigue profiles (*P* < 0.001, *η*^*2*^ = 0.263), as detailed in Table 4. Post hoc tests showed that class 1 had the lowest score, followed by class 2, while class 3 had the highest score. All comparisons were statistically significant (*P* < 0.001). In addition to one-way ANOVA, the BCH method was also used to verify the research findings. As shown in Table 5, the results of the BCH method are consistent with those of the one-way ANOVA.

**Table 4:** Differences in work alienation among CNPs with different latent profiles of compassion fatigue (*n* = 320)Note: data are Mean ± SD. ^a^ compared with the class 1, *P* < 0.05. ^b^ compared with the class 2, *P* < 0.05. class 1 = high-satisfaction-low-exhaustion group; class 2 = moderate compassion fatigue group; class 3 = severe exhaustion group.

Variable	Class 1 (*n* = 56)	Class 2 (*n* = 160)	Class 3 (*n* = 104)	F value	*P* value	Post hoc tests
Sense of powerlessness	6.61 ± 1.56	13.07 ± 2.51^a^	16.05 ± 2.60^ab^	37.721	< 0.001	Class 1 < Class 2 < Class 3
Sense of helplessness	13.45 ± 2.11	12.48 ± 2.22 ^a^	10.78 ± 2.37 ^ab^	31.542	< 0.001	Class 1 > Class 2 > Class 3
Sense of meaninglessness	10.39 ± 2.90	14.04 ± 3.11 ^a^	19.76 ± 2.75 ^ab^	209.253	< 0.001	Class 1 < Class 2 < Class 3
Overall	24.05 ± 2.42	36.98 ± 3.72 ^a^	47.72 ± 3.84 ^ab^	56.419	< 0.001	Class 1 < Class 2 < Class 3

**Table 5:** Comparison of work alienation among the three latent Profiles. *** *P* < 0.05.

Group	Class 1	Class 2	Class 3	Overall chisquare test
Class 1	0			
Class 2	58.79^***^	0		125.08^***^
Class 3	102.78^***^	52.89^***^	0	

### Multinomial logistic regression analysis of the influencing factors of different latent profiles of compassion fatigue among CNPs

After conducting a univariate analysis, variables with statistical significance were evaluated for multicollinearity. The VIF values varied from 1.645 to 4.433, indicating no multicollinearity among the variables and strengthening the robustness of the analytical model. Variables demonstrating statistical significance in the univariate analyses (age, marital status, education, years of preceptorship experience, employment type, and professional title) were incorporated as independent variables in a multinomial logistic regression model, with the three latent compassion fatigue profiles specified as the dependent variable. The coding scheme for the independent variables is shown in Table 6.

The factors influencing compassion fatigue among the CNPs with different latent profiles are listed in Table 7. Compared to Class 3, Class 1 CNPs had a greater tendency to be primary nurses aged 29 years or younger, with 2–5 years of teaching experience and holding a formal position. Those unmarried or divorced CNPs with a junior college education were more likely to be classified in class 3. In contrast to Class 3, Class 2 CNPs had a greater tendency to report being primary nurses who were 29 years old or younger, had 5–10 years of teaching experience, and held a formal position. However, unmarried or divorced CNPs aged 30–39 with junior college education were more likely to be classified into class 3.

**Table 6:** The method of assigning values to independent variables

Variable	coding scheme
Age	≤ 29 = 1; 30–39 = 2; ≥ 40 = 3;
Years of preceptorship experience	2–5 = 1; 6–10 = 2; > 10 = 3;
Marital status	unmarried = 0, married = 1;
Education	Junior college = 0, Bachelor’s degree or above = 1;
Employment type	Permanent staff nurse = 0 , Contract dispatch or others = 1;
Professional title	Primary Nurse = 0 , Nurse-in-Charge and above = 1;

**Table 7:** Multinomial logistic regression analysis of different profiles.Reference group: class 3 = severe exhaustion group. OR, odds ratio; 95% CI, 95% confidence Interval.

Group	Variable	β	Sb	OR	95% CI	*P*
**Class 1 VS Class 3**	Age (years) ≤ 29 (ref.: ≥ 40)	3.766	0.912	43.2	(7.23, 58.15)	< 0.001
	30–39 (ref.: ≥ 40)	-1.204	0.888	0.30	(0.05, 1.71 )	0.175
	**Marital status** Unmarried or divorced **(**ref.: Married)	-2.120	0.376	0.12	(0.06, 0.25)	< 0.001
	**Education** Junior college **(**ref.: Bachelor’s degree or above)	-2.232	0.381	0.11	(0.05, 0.23)	< 0.001
	**Years of preceptorship experience**					
	2–5 (ref.: > 10)	2.315	0.501	10.13	(3.79, 27.03)	< 0.001
	6–10 (ref.: > 10)	1.011	0.467	2.75	(1.10, 6.86)	0.030
	**Employment type**					
	Permanent staff nurse (ref.: Contract dispatch or others)	3.233	0.441	25.36	(10.69, 60.18)	< 0.001
	**Professional title** Primary Nurse (ref.: Nurse-in-Charge and above)	2.015	0.373	7.50	(3.61, 15.57)	< 0.001
**Class 2 VS Class 3**	**Age (years)** ≤ 29 (ref.: ≥40)	1.376	0.608	3.96	(1.20, 13.03)	0.024
	30–39 (ref.: ≥40)	-1.139	0.418	0.32	(0.14, 0.73)	0.006
	**Marital status** Unmarried or divorced (ref.: Married)	-1.329	0.282	0.27	(0.15, 0.46)	< 0.001
	**Education** Junior college (ref: Bachelor’s degree or above) **Years of preceptorship experience (years)**	-1.567	0.288	0.21	(0.12, 0.37)	< 0.001
	6–10 (ref.: > 10)	1.095	0.306	2.99	(1.64, 5.45)	< 0.001
	**Employment type** Permanent staff nurse (ref.: Contract dispatch or others)	2.263	0.346	9.61	(4.88, 18.92)	< 0.001
	**Professional title** Primary Nurse (ref.: Nurse-in-Charge and above)	1.249	0.276	3.49	(2.03, 5.99)	< 0.001

## Discussion

### Latent profiles of CNPs’ compassion fatigue and their characteristics

Three latent compassion fatigue profiles were identified among the CNPs: high-satisfaction-low-exhaustion group, moderate compassion fatigue group, and severe exhaustion group. Among them, the high-satisfaction-low-exhaustion group accounted for 17.5%. This group was characterised by high compassion satisfaction, low burnout levels, and mild secondary traumatic stress. The group’s demographic characteristics included being young, married, having higher levels of education, less teaching experience in clinical settings, junior professional titles, job stability, and higher income levels. Their work attitude was described as positive and enthusiastic, with less frequent experiences of burnout and trauma. However, this positive profile is nuanced by a significant sense of helplessness, which, as will be discussed, likely reflects their junior status within the healthcare system rather than a lack of idealism. These findings align with the systematic review by Alharbi et al.^24^; however, their work focused specifically on ICU nurses, whereas the present study examined general CNPs and arrived at comparable conclusions. Another cross-sectional study involving Saudi nurse preceptors^25^ demonstrated that compassion satisfaction was significantly and inversely related to burnout and secondary traumatic stress. Enhancing compassion satisfaction can directly improve job satisfaction, thereby maintaining enthusiasm for work and reducing turnover intention. In this study, the high satisfaction-low exhaustion group also exhibited similar characteristics. The CNPs in the moderate compassion fatigue group accounted for 50% of the total sample. Their average scores on the scale as well as scores in each dimension remained at a moderate level, reflecting a stable overall state. This finding suggests that moderate compassion fatigue is the main form of compassion fatigue among CNPs. In this group, the score for the compassion satisfaction dimension was slightly higher than that for burnout and secondary traumatic stress, indicating that the CNPs were still able to maintain a certain level of enthusiasm in clinical work. However, there is still potential for further improvement in burnout and secondary traumatic stress. The proportion of the severe exhaustion group accounts for 32.5%, distinguished by severe burnout and secondary traumatic stress, alongside reduced compassion satisfaction. According to Yu et al.^26^, oncology nurses also exhibit a subgroup characterized by “high-traumatic-stress, high-burnout, low- compassion-satisfaction” in terms of compassion fatigue. This aligns with the features of the severe exhaustion group in this study, indicating that special attention should be given to nurse preceptors in this group.

Therefore, nursing managers should implement tailored strategies based on the unique characteristics of each group of nurses.

- For the severe exhaustion group, immediate intervention is critical. Research indicates that insufficient social support is a primary factor linked to burnout^27^. Nursing managers should initiate regular, structured, one-on-one conversations to evaluate workload appropriateness and provide psychological support. Organizing department-led team-building activities, such as outdoor excursions or wellness retreats, can help alleviate occupational burnout and mitigate CF symptoms^28,29^.
- For the moderate compassion fatigue group, the focus is on proactive prevention. Establishing peer-led support groups for regular emotional exchange is recommended to maintain work enthusiasm. Simultaneously, providing clear career development planning and guidance can prevent the deterioration of CF.
- For the high-satisfaction-low-exhaustion group, the strategy is to sustain and empower. To maintain their positive state and address their underlying helplessness, managers can offer them opportunities to lead small quality improvement projects or act as mentors for new nurses. This not only enhances their sense of professional belonging but also provides them with a tangible way to influence their work environment.

### Multinomial logistic regression analysis of the influencing factors of compassion fatigue in different latent profiles of CNPs

The multinomial logistic regression analysis in this study revealed a clear “job role demands–resources” dual-pathway model: compared to those aged ≥ 40 years, CNPs under 29 years of age were significantly more likely to fall into the “high-satisfaction-low-exhaustion” profile (C1 vs. C3: *OR* = 43.20, *P* < 0.001) and the “moderate compassion fatigue” profile (C2 vs. C3: *OR* = 3.96, *P* = 0.024);the 30–39 age group showed a significantly lower (*OR* = 0.32, *P* = 0.006) in the comparison between the moderate compassion fatigue group and the severe exhaustion group, indicating a tendency for this group to cluster more in the severe exhaustion profile. This aligns with our interpretation of the 30–39 age range as the “critical period of overlapping role responsibilities.” Previous studies on the relationship between age and CF have yielded inconsistent conclusions^30,31^, and the discrepancies may be related to the limited scope of single-department samples and broad age groupings of participants. This study adopted a more refined stratification and covered multiple departments throughout the hospital, which helped reveal the motivational pathways activated by initial professional motivation and team learning support—personal/work resources—during the early stages of a professional career. When CNPs aged 30–39 face overlapping demands from promotion, research, teaching, and family, they experience a lag in recovery and resource replenishment, which leads them into a path of health depletion^32,33^. In terms of marital status, the likelihood of unmarried/divorced individuals entering the C1 and C2 was significantly reduced.

(C1 vs. C3: *OR* = 0.12; C2 vs. C3: *OR* = 0.27; both *P* < 0.001), suggesting that the social resources provided by stable partner support have a buffering effect on secondary trauma intrusion and emotional exhaustion^34^. In terms of education, having an associate degree, compared to a bachelor’s degree or above, significantly reduced the likelihood of being classified as C1 (*OR* = 0.11, *P* < 0.001) and C2 (*OR* = 0.21, *P* < 0.001), which is consistent with findings that higher levels of education promote compassion satisfaction and reduce burnout and secondary trauma^35,36^. This reflects the key role of the “cognitive/professional resource matrix” formed by critical thinking, evidence-based responses, and professional competence in balancing resources and demands. Regarding employment type, CNPs significantly increased the likelihood of entering C1 (*OR* = 25.36, *P* < 0.001) and C2 (*OR* = 9.61, *P* < 0.001), indicating that structural security and organizational embeddedness can activate motivational pathways by enhancing identity and psychological safety^37^.

In terms of professional titles, junior professional titles were significantly more likely to be classified as C1 (*OR* = 7.50, *P* < 0.001) and C2 (*OR* = 3.49, *P* < 0.001) compared to intermediate and higher titles, which is consistent with evidence that higher professional titles are associated with increased decision-making complexity, emotional and cognitive demands, and greater responsibility-related stress^38^. As for the duration of preceptorship for CNPs, it exhibited a “reverse U-shape” or “mid-career advantage” pattern: using > 10 years as the reference, 2–5 years (C1 vs. C3: *OR* = 10.13, *P* < 0.001) and 6–10 years (C1 vs. C3: *OR* = 2.75, *P* = 0.030; C2 vs. C3: *OR* = 2.99, *P* < 0.001) of precepting experience were associated with a lower likelihood of entering the Class3 profile. This suggests that during the intermediate period, personal resources such as self-efficacy and a sense of subjective control, formed by “proficient skills + sustained freshness,” are sufficient to offset clinical and emotional demands of the job. However, with > 10 years of experience, “resource erosion/accumulated fatigue” and emotional regulation exhaustion may emerge, with health deterioration becoming the predominant pathway. Overall, the results display a “dual-pathway” integration under the JD-R framework: one is the “Role and Responsibility Intensification” chain (ages 30–39, higher professional titles, > 10 years of teaching experience), corresponding to accumulated decision-making complexity and emotional and cognitive exhaustion; the other is the “Multi-layered Resource Buffer” chain (married/partner support, cognitive and professional resources brought by higher education, structural security from formal employment, sense of efficacy and control during 2–10 years of teaching, and the initial professional motivation characteristic of early careers), which strengthens motivation and recovery. Years of teaching experience showed a pattern of “mid-phase protection, long-term erosion,” suggesting a dynamic balance between gains from experience and resource depletion. Overall, the results support the notion that “insufficient resource combination and accumulated role demands” is the core pathway for CNPs entering a state of severe exhaustion. It is recommended that healthcare administrators intervene early in their careers, such as through flexible scheduling, regular psychological counseling, and organizational stress-relief activities, to prevent the progression toward high exhaustion trajectories.

### Significant differences in work alienation across compassion fatigue latent profiles

This study shows that there are significant gradient differences in work alienation among the latent profiles of CF: overall work alienation *F*_(2,317)_ = 56.419, *P* < 0.001, *η* = 0.263. The total work alienation score of the severe exhaustion group (47.72 ± 3.84) was nearly twice that of the high-satisfaction-low-exhaustion group (24.05 ± 2.42), The dimensions of powerlessness and meaninglessness increased progressively with the degree of exhaustion (Class 1 < Class 2 < Class 3). However, the “sense of helplessness” dimension revealed a paradoxical trend, being highest in the high-satisfaction-low-exhaustion group (Class 1 > Class 2 > Class 3). This finding suggests that this group’s satisfaction is not rooted in idealism but in their direct clinical role, while their helplessness stems from structural constraints. As junior-ranking CNPs, they feel effective in their immediate duties but lack the autonomy to influence broader systemic issues, thus feeling helpless to change their work environments. Conversely, the lowest helplessness score in the severe exhaustion group likely signifies defensive detachment, rather than empowerment. Prolonged burnout can foster disengagement, where individuals cease striving for change, extinguishing their sense of helplessness. This phenomenon aligns with the characteristics of automatic defense mechanisms and empathy contraction triggered by emotional exhaustion. From the perspective of the JD-R model, high emotional labor exposure (pain, trauma, death scenarios) and a high density of uncertain events constitute the ongoing “job demands”side, while insufficient timely psychological support, emotional debriefing, and resource replenishment result in a relative lack on the “job resources” This simultaneously drives emotional exhaustion in the energy channel and the collapse of meaning in the motivation channel, which in turn triggers work alienation as a manifestation of “defensive withdrawal”.^39^ Previous studies have shown that there may be a bidirectional reinforcement between emotional exhaustion and work alienation: exhaustion weakens intrinsic motivation and role identification, deepening alienation; meanwhile, work alienation reduces emotional and cognitive engagement, accelerating the spiral of exhaustion^40,41^. Emotional exhaustion is not only the core of burnout but also an important risk factor for CF^42^. Therefore, the differences in work disengagement observed across different profiles reflect the stratified state of cumulative resource depletion. CNPs are subjected to high workloads and occupational hazards over extended periods (night shifts, teaching duties, conflicts, end-of-life events)^43^, making them more prone to entering a resource-loss spiral. Without timely compensation, this may push those in the “high satisfaction-low-exhaustion” or “moderate compassion fatigue” profiles toward the “severe exhaustion” profile, intensifying feelings of work disengagement. An increase in work alienation is associated with negative occupational outcomes, such as reduced productivity, decreased motivation and commitment, lower job satisfaction, and higher intentions to leave the job^44,45^. It can also lead to strained nurse-patient relationships, nursing errors, diminished overall quality of care, and ultimately a reduction in patient safety and experience^46^. Therefore, in practice, it is necessary to both “reduce demand and supplement resources”: dynamically optimize the allocation of highly traumatic situations and shift structures to limit emotional load peaks; establish regular psychological support and mindfulness-based stress reduction (MBSR) interventions to enhance emotional regulation and recovery abilities;^47^ build a multidimensional emotional support system (such as mentorship programs, peer pairing, and structured emotional debriefings);^48^ and incorporate targeted screening and indicators of work alienation into routine monitoring to form early warnings and interrupt the negative cycle of compassion fatigue—work alienation.

## Conclusion

In this study, the use of LPA based on CNPs’ compassion fatigue scores identified three latent profiles: high-satisfaction-low-exhaustion group, moderate compassion fatigue group, and severe exhaustion group. These findings indicate that compassion fatigue is prevalent among CNPs and exhibits marked heterogeneity. We further identified that CNPs with different CF profiles showed statistically significant differences across work alienation. As the level of CF deepens, the sense of work alienation among the CNPs becomes more pronounced. Our findings suggest that personalised interventions could be developed for nurse preceptors experiencing different types of CF during clinical practice to strengthen the guidance of professional identity, alleviate the degree of compassion fatigue, reduce their sense of work alienation, and decrease nursing staff turnover, thereby helping to improve the quality of nursing services. These results may provide theoretical and empirical support for clinical managers and public health professionals to implement targeted, stratified interventions. While the latent profile analysis employed in this study reveals important heterogeneity in CNPs’ compassion fatigue, its cross-sectional design and single-center sample underscore the necessity for future longitudinal and multi-center research to verify and build upon these findings.

### Limitation

Several limitations should be noted. First, this cross-sectional investigation recruited hospital CNPs via convenience sampling. As a non-probability sampling method, this approach, combined with the inclusion of nursing staff from only one tertiary grade A general hospital, may introduce a selection bias and limit the generalisability of our findings to the broader nursing population. Second, all data were collected via self-reported questionnaires, which may be subject to recall or social desirability bias. Third, although we adjusted for several covariates, other unmeasured factors (e.g., family support and detailed workload intensity) might also influence compassion fatigue and work alienation.

### Implications and future research directions

Our study identified heterogeneity in CNPs’ compassion fatigue and outlined the current state of work alienation in a tertiary Grade A general hospital in China, providing nursing managers with a theoretical foundation for developing targeted interventions. We used LPA to identify different latent profiles of CF and their characteristics, and examined the associations between nurse preceptors with different latent profiles and multiple dimensions of work alienation. Previous studies^1,6,14^have mostly focused on the negative effects of compassion fatigue, such as occupational burnout and work alienation. Limited research has used LPA to categorise CNPs’ compassion fatigue and analyse its relationship with work alienation. This study addresses a gap in the literature and provides evidence-based insights that may inform management strategies, guide future interventions, and potentially contribute to reducing adverse events and enhancing the quality of nursing care. Ultimately, it is designed to reduce CF and work alienation in nursing staff and lower staff turnover, and enhance patient satisfaction with nursing care, thereby promoting a positive cycle in the nursing work. The cross-sectional design precludes causal inference, and convenience sampling limits the generalizability of the findings. To mitigate these limitations, future research should prioritise conducting large-sample, multicentre longitudinal studies and include more variables to verify the viewpoints of this study.

## Data Availability

Public sharing of our data may be subject to ethical and legal restrictions. Researchers wishing to access our data can contact the corresponding author at 540605689@qq.com.
